# Effects of Topography and Extracellular Matrix Composition on Focal Adhesion Patterning in Human Corneal Fibroblasts

**DOI:** 10.3390/ijms262411935

**Published:** 2025-12-11

**Authors:** Divya Subramanian, Nathaniel S. Tjahjono, Tarik Z. Shihabeddin, Satweka Nammi, Miguel Miron-Mendoza, Victor D. Varner, W. Matthew Petroll, David W. Schmidtke

**Affiliations:** 1 Department of Bioengineering, University of Texas at Dallas, Richardson, TX 75080, USA; divya.subramanian@utdallas.edu (D.S.); nathaniel.tjahjono@utdallas.edu (N.S.T.); tarik.shihabeddin@utdallas.edu (T.Z.S.); satweka.nammi@utdallas.edu (S.N.); vdv@utdallas.edu (V.D.V.); 2 Department of Ophthalmology, University of Texas Southwestern Medical Center, Dallas, TX 75390, USA; miguel.miron@utsouthwestern.edu (M.M.-M.); matthew.petroll@utsouthwestern.edu (W.M.P.); 3 Department of Biomedical Engineering, University of Texas Southwestern Medical Center, Dallas, TX 75390, USA

**Keywords:** cornea, focal adhesions, collagen fibrils, topography, corneal fibroblasts, micropatterns, integrins

## Abstract

Corneal fibroblasts adhere to the extracellular matrix via integrin-containing focal adhesions (FAs). Although topographical cues are known to influence FA patterning in corneal fibroblasts, it is unclear how ECM composition, biophysical cues, and specific integrins modulate FA patterning in corneal fibroblasts. In this study, we cultured a human corneal fibroblast cell line (HTKs) on different ECM proteins and micropatterns of aligned collagen fibrils to determine the effects of ECM topography and composition on focal adhesion subcellular patterning. Using confocal imaging, we observed and quantified changes in FA and integrin patterning based on the underlying ECM type. More specifically, the presence of fibrillar topography as compared to monomeric collagen resulted in diminished FA number, area, and length. Using specific integrin blocking antibodies, we also demonstrate that HTKs use different integrin subunits to adhere to specific ECM coatings. For example, β_1_ integrins are important in adhesion formation when corneal fibroblasts adhere to collagen, while α_5_ integrin is important for the HTKs to adhere to fibronectin. Blocking of α_5_ integrin did not completely inhibit cell spreading and FA patterning when cells adhered to fibronectin. These results suggest that there might be other fibronectin receptors that HTKs use in the absence of α_5_ integrin. These results lay the foundation to understand the role of different integrin subunits in FA patterning. Through further experimentation using our developed platform, we envision that a better understanding of the integrins and their associated signaling could have implications for advanced in vitro and in vivo applications in cornea biology.

## 1. Introduction

The extracellular matrix (ECM) contains a wealth of biochemical, mechanical, and topographical cues, and cellular interaction with the ECM plays a key role in a number of physiological and pathophysiological processes such as cell proliferation, differentiation, migration, fibrosis, and apoptosis. A key regulator of cell–ECM interactions is the integrins, which are transmembrane receptors that mediate cell interactions with ECM glycoproteins (e.g., collagen, fibronectin) and mechanically link the ECM to the cell’s actin cytoskeleton [1]. Initial binding of an integrin receptor to an ECM protein can be relatively weak. However, the initial binding frequently leads to the recruitment and lateral clustering of additional integrins and can eventually lead to the formation of more stable focal adhesions (FAs) and the assembly of actin stress fibers [2]. Integrin-mediated focal adhesions play an important role in cellular mechanosensing, induce several intracellular processes, and are sensitive to the ECM composition and architecture [3,4].

In several organs and tissues, ECM proteins are arranged in distinct spatial patterns that impact not only the structure and topography of these tissues but also their function. For example, in tendons, Type I collagen is organized as aligned collagen fibrils that are parallel to the tendon’s long axis and provide the tendon’s strength [5]. Similarly, in the cornea, aligned Type I collagen fibrils are organized into lamellae, which are essential for the transparency of the cornea [6,7]. Studies of cells cultured on 2D and in 3D aligned matrices have reported that the orientation of aligned structures (e.g., fibrils and fibers) can not only modulate the interaction with integrins but also the adhesive behavior (e.g., polarization, orientation, migration, directional persistence, spreading) of cells via several mechanisms. For example, it has been reported that aligned collagen fibers provide greater resistance to cell pulling than nonaligned fibers, resulting in fewer and longer protrusions [8]. Similarly, a relationship between fiber orientation and adhesion maturation was reported, where aligned fibers promoted longer adhesions and an increased rate of adhesion elongation as compared to random fiber orientations [4].

Although there are several reports on how ECM composition [9,10,11,12,13] and topography [14,15,16,17,18] can influence corneal cell behavior (e.g., contact guidance, cell activation, cell alignment), how the ECM composition and topography impact FA formation and localization, as well as integrin spatial patterning in corneal cells, is not fully understood. Initial studies on FAs have been generally qualitative in nature, while recent papers have attempted to quantify these interactions. For example, Zhurenkov and colleagues cultured human corneal stromal fibroblasts on two fibrillar substrates, decellularized human amniotic membrane (dHAM) and films of random fibrillar collagen, in the presence of 10% FBS and observed that FAs were more frequent and larger on random collagen fibrils as compared to tissue culture plastic (TCPS) [19]. Similarly, Bhattacharjee and colleagues cultured human corneal keratocytes (HCKs) on uncoated polydimethylsiloxane (PDMS) substrates that contained line, lattice, and micropit topographies and observed increased FA areas on the lattice and micropit patterned substrates [20]. When human corneal stromal cells were cultured on PDMS substrates containing microgrooves, FAs on patterned surfaces were longer, located along the top of ridges, and directed in the groove direction, while FAs on plain surfaces lacked any defined orientation [21]. Finally, Drier and colleagues cultured immortalized human corneal epithelial cells (hTCEpi) on topographical patterns of grooves and ridges that were coated with a serum-free tissue culture reagent containing fibronectin, collagen, and albumin (FNC coating mix). Although there were no significant changes in the expression of FAK on the topographically patterned surfaces, they did observe an increased percentage of aligned cells after FAK knockdown and decreased rates of cell migration [22]. In this study, we investigated the effects of biophysical (i.e., topography) and biochemical (different ECM proteins) cues on focal adhesion formation and integrin binding in corneal fibroblasts by culturing cells on different ECM protein coatings. We observed that the type of ECM coating influenced the number and size of focal adhesions that were formed by the corneal fibroblasts. We also observed the role of different integrin subunits in focal adhesion formation through integrin-blocking experiments.

## 2. Results and Discussion

### 2.1. Deposition of Aligned and Random Collagen Fibrils on PDMS-Coated Glass Coverslips

We have previously shown that aligned collagen fibrils can be deposited on PDMS-coated glass coverslips due to the hydrophobic nature of PDMS [23,24]. In this study, we patterned 750 µm wide aligned collagen fibrils on PDMS-coated cover slips. Random fibrillar collagen was deposited as outlined previously [23]. We chose aligned and randomly orientated collagen since the corneal stroma consists of type-I aligned fibrils of varying widths (0.2–250 μm), which can be disrupted in wound healing, resulting in irregular or randomly oriented fibril deposition by the keratocytes. The fibrils were stained with DTAF and imaged using a Confocal Microscope (Supplementary Figure S1A). A dense mat of collagen fibrils that consisted of both short and long fibrils aligned in the direction of flow (horizontal) was observed. Random collagen fibrils stained with DTAF were also imaged and showed uniform deposition and no preferred directional orientation (Supplementary Figure S1B).

### 2.2. ECM Composition and Alignment Modulate Focal Adhesion Patterning

**Figure 1:**
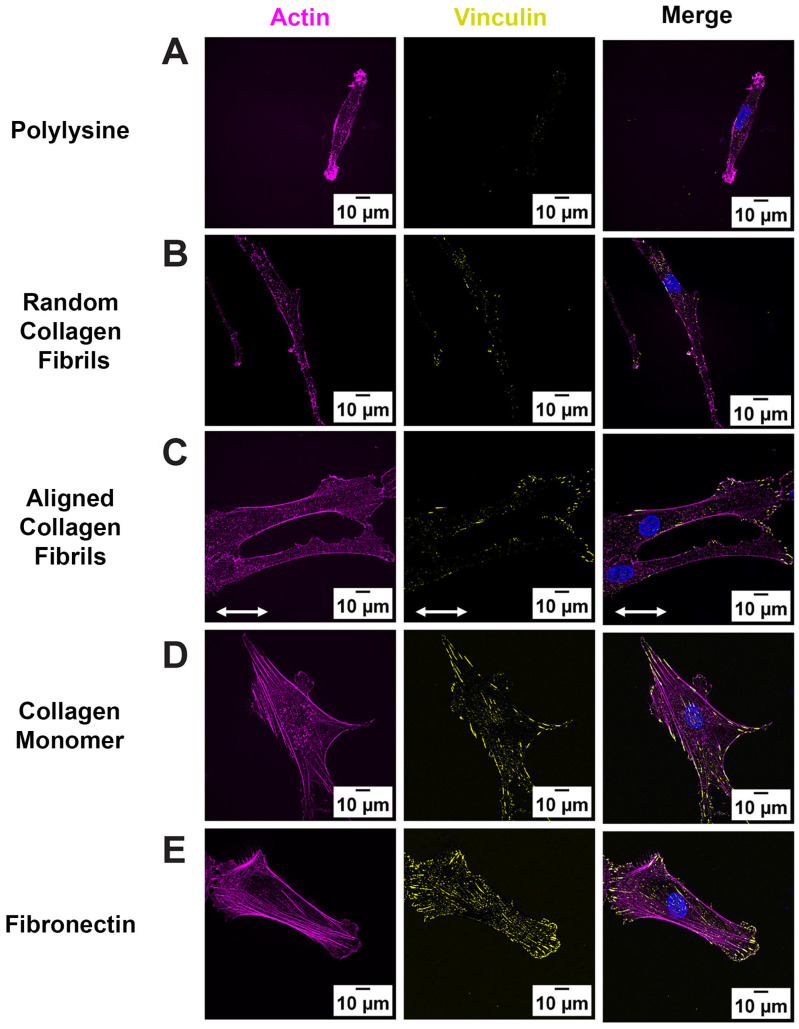
Fibril Topography regulates FA formation: Representative fluorescent images of HTK cells cultured on homogeneous coatings of (**A**) Poly-lysine, (**B**) Random Fibrils, (**C**) Aligned Collagen Fibril Micropattern, (**D**) Collagen Monomer, and (**E**) Fibronectin-coated surfaces. Cells were stained for Vinculin (Yellow), Phalloidin (Magenta) and Nuclei (Blue). Scale Bar = 10 μm. White double arrow indicates fibril direction, and fibrils are oriented horizontally.

Previous studies have shown that topographical cues can modulate focal adhesion subcellular patterning in corneal keratocytes and fibroblasts [14,19,21]. However, few studies have examined how different ECM proteins or the alignment of collagen fibrils modulate FAs in corneal cells. Given that FA size impacts cell migration [25,26], cell morphology [19,25,27], and adhesion strength [28,29], we investigated the role of ECM type and fibrillar topography on FA formation, by culturing human corneal fibroblasts (HTKs) on 5 different substrates: monomeric collagen, aligned collagen fibrils, randomly oriented collagen fibrils, fibronectin, and Poly-L-Lysine (PLL) for 24 h and immunofluorescently labeled for vinculin (Figure 1). PLL-coated substrates were included to determine if non-specific adhesion modulated FA patterning in HTKs. FAs were detected on all surfaces except on PLL surfaces, where FA formation was absent (Figure 1A). No detection of FAs for HTKs cultured on PLL was not surprising, given that cell adhesion to PLL is integrin independent and does not lead to focal adhesion formation [30,31]. In contrast, HTKs cultured on surfaces coated with monomeric collagen and fibronectin showed large FAs that were distributed throughout the cell and colocalized with the actin stress fibers (Figure 1D,E). HTKs cultured on substrates with topography (i.e., aligned and randomly oriented collagen fibrils) showed smaller FAs that were primarily localized along the cell periphery (Figure 1B,C).

**Figure 2:**
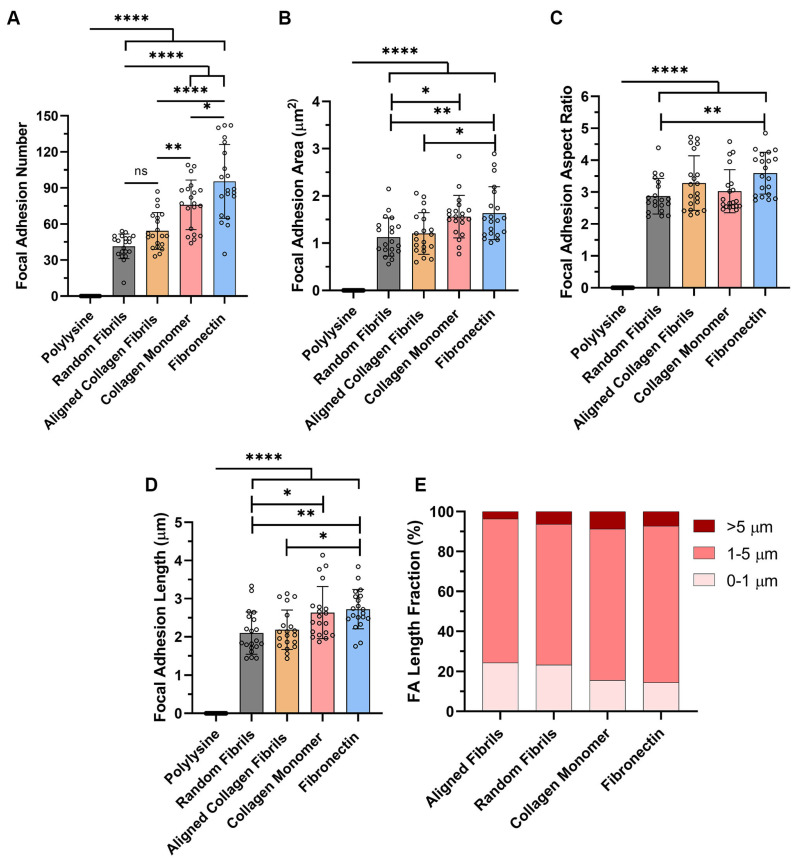
Effects of Topography and ECM Composition on FA Morphology. Effect of substrate coating on (**A**) number of FAs per cell; (**B**) FA area; (**C**) FA aspect ratio; (**D**) FA length; and (**E**) types of FA formed. Data represents Mean ± Standard Deviation for 4 experimental repeats and a total of 20 cells were analyzed. Each data point represents average values for a single cell. ns *p* > 0.05; * *p* < 0.05; ** *p* < 0.01; **** *p* < 0.0001.

Since FAs develop from small circular dots into elongated shapes as they mature [32], we used confocal imaging to analyze and quantify differences in the number and morphology of FAs on the different surface coatings. As shown in Figure 2A, the number of FAs formed was significantly lower on the substrates containing topography (i.e., random and aligned collagen fibrils), as compared to the flat substrates coated with monomeric collagen or fibronectin. Likewise, there were significant differences in FA Area and length on substrates with topography vs. the smooth protein-coated substrates (Figure 2B,D), with the largest focal adhesion areas and lengths being observed on fibronectin. Interestingly, despite differences in area and length, the FA aspect ratio (AR), which is an indicator of the elongated FAs, remained relatively constant (AR ~3) on most of the surface coatings (Figure 2C). It should be noted that there were significant differences in the AR between random fibrils and fibronectin-coated surfaces.

To further investigate subtle changes in focal adhesions on the different substrates, we initially created cumulative frequency plots for FA area, length and aspect ratio (Supplemental Figure S2A–C). The cumulative frequency plots for FA area and FA length showed that the populations of FA on the substrates with topography (i.e., random and aligned collagen fibrils) were left-shifted toward smaller FA sizes as compared to the substrates without topography (i.e., collagen monomer and fibronectin). The cumulative frequency plot of the focal adhesion aspect ratios showed only a distinguishable difference for the focal adhesions formed on fibronectin.

Previous studies have suggested that FAs can be classified as focal complexes (<1 μm), mature FAs (1–5 μm) or supermature FAs (>5 μm) based on their lengths [33,34]. Thus, we performed a similar analysis (Figure 2E) and observed that the primary structure that was formed on all the substrates was mature FAs, accounting for 70–78%. However, there were differences in the percentage of focal complexes and supermature FAs generated on the different substrates. On substrates coated with either aligned or random collagen fibrils the percentage of focal complexes formed (~24%) was 60% higher than the focal complexes formed on the smooth collagen (15%) and fibronectin (14.5%). Conversely, HTKs formed ~2 times less supermature FAs on aligned collagen fibrils (3.6%) then on random fibrils (6.3%), collagen monomer (8.7%) or fibronectin (7.2%).

Our observations of a reduced number and size of FAs on fibrillar (aligned and random) collagen are consistent with previous studies in corneal fibroblasts, which reported that nanoscale topography cues caused fewer and smaller FAs [14,21]. Although a previous study has reported that corneal fibroblasts prefer to adhere to fibronectin over other ECM proteins (i.e., Type I collagen, vitronectin) [35], our observation of an increase in FA number and FA size when corneal fibroblasts were cultured on fibronectin-coated surfaces is novel. Likewise, our analysis and classification of FAs as nascent, mature, and supermature reveal new insights as to the types of focal adhesions formed by corneal fibroblasts in response to topography and ECM composition. The reduced formation of focal adhesions that was observed on substrates with topographies may be relevant to reports that topography can inhibit differentiation of corneal fibroblasts into myofibroblasts [16].

### 2.3. Corneal Fibroblasts Expression of Integrin α_5_ and β_1_ on Different ECM Coatings

**Figure 3:**
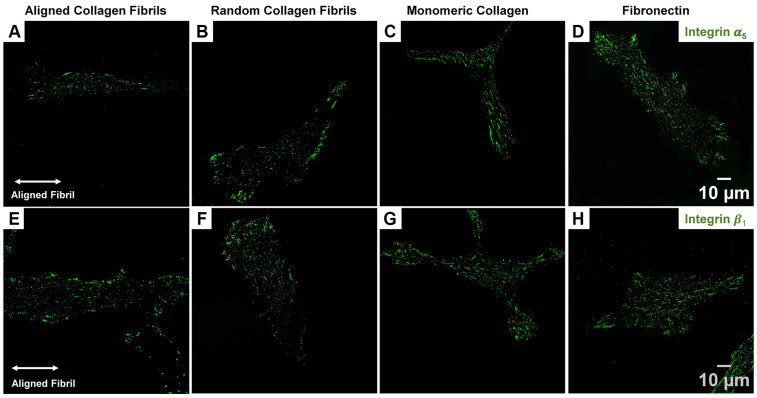
Effect of ECM coating type on α_5_ and β_1_ Integrin expression in HTK cells. Representative fluorescent images for α_5_ (**A**–**D**) and β_1_ (**E**–**H**) staining for HTKs cultured on Aligned Collagen Fibrils, Random Fibrils, Collagen Monomer, and Fibronectin-coated surfaces. Scale Bar = 10 μm.

Previous studies have shown that corneal fibroblasts express the well-known fibronectin receptor α_5_β_1_ integrin [35,36,37]. Similarly, our group has shown that blocking the α_5_ subunit results in cytosolic YAP retention and that HTKs secrete fibronectin when cultured on different ECM coatings [24]. Since focal and fibrillar adhesions are associated with integrin-mediated adhesion (e.g., α_v_β_3_ and α_5_β_1_ integrins), we investigated the expression pattern of α_5_ and β_1_ by immunostaining of HTKs cultured on different ECM coatings. On fibrillar substrates (aligned and random collagen), we observed that both the α_5_ (Figure 3A,B) and β_1_ (Figure 3E,F) subunits exhibited primarily small punctate clusters distributed throughout the cell, with a few elongated punctate clusters localized to the cell periphery. In contrast, on the smooth protein-coated surfaces (i.e., monomeric collagen and fibronectin), we observed an increased number of elongated α_5_ (Figure 3C,D) and β_1_ (Figure 3G,H) punctate distributed throughout the cell. This expression of integrin subunits mirrored the FA vinculin staining focal adhesion data of Figure 1.

### 2.4. Focal Adhesion Patterning Is Regulated by Integrin Subunits and ECM Composition

It has been suggested in other cell types that focal adhesion formation, maturation and patterning are regulated by a multifaceted interplay between biochemical (e.g., ECM composition) and biophysical (i.e., topography, elasticity) cues. As shown in Figure 1 and Figure 3, the HTKs displayed distinct patterns of FAs and the integrin subunits on the different ECM coatings. The results in Figure 2 also showed that FA subcellular patterning varied with the underlying ECM composition and agree with reports that ECM composition primarily determines integrin binding specificity. To directly determine which integrin subunits were responsible for focal adhesion subcellular patterning, HTKs were seeded on aligned collagen fibrils, random fibrils, monomeric collagen-coated, and fibronectin-coated substrates in the presence of α_5_ and β_1_ blocking antibodies. Cells cultured in the absence of function-blocking antibodies were used as controls.

**Figure 4:**
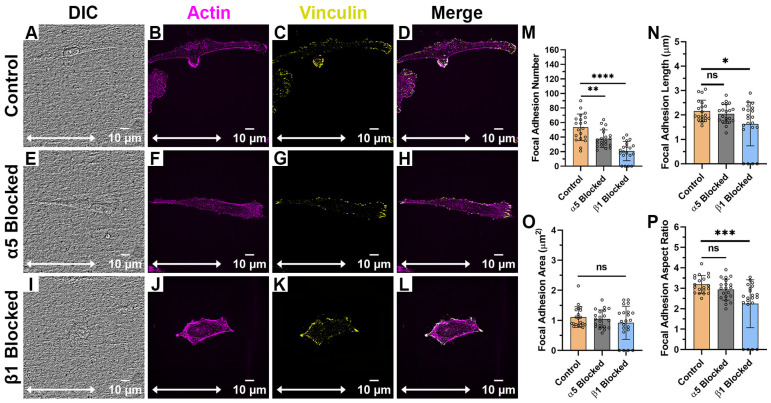
Effect of Integrin Blocking on Focal Adhesion Patterning on Aligned Fibril Substrates: Confocal Images for cells cultured on Aligned Collagen Fibril Substrates without Integrin Blocking (Control) (**A**–**D**), with α_5_ blocking antibody (**E**–**H**) and with β_1_ function blocking antibody (**I**–**L**). Quantification of Focal Adhesion morphologies with and without integrin blocking (**M**–**P**). White double arrow indicates fibril direction, and fibrils are oriented horizontally. Cells were labeled for Vinculin (Yellow) and Actin (Magenta). Scale Bar = 10 μm. Data represents Mean ± Standard Deviation for 4 experimental repeats and a total of 20 cells were analyzed. Each data point represents average values for a single cell. ns *p* > 0.05; * *p* < 0.05; ** *p* < 0.01; *** *p* < 0.001; **** *p* < 0.0001.

When HTKs were cultured on the aligned collagen fibril substrates (Figure 4A–L), there was little to no reduction in cell spreading area when the α_5_ subunit was blocked (Figure 4E–H). However, when the β_1_ subunit was blocked, there was a significant reduction in the cell spreading area with the cells adopting a more rounded and non-polarized morphology (Figure 4I–L). Blocking of the α_5_ subunit resulted in a 30% reduction in the number of focal adhesions (Figure 4M, Table 1) but did not significantly change the FA length (Figure 4N), FA area (Figure 4O), or FA aspect ratio (Figure 4P). In contrast, when the β_1_ integrin was blocked, there was not only a 61% reduction in the number of focal adhesions formed (Figure 4M), but there was also a significant reduction in the FA Length (Figure 4N) and FA Aspect Ratio (Figure 4P). It should be noted that blocking β_1_ caused some cells to exhibit no focal adhesions. The reduction in cell area and focal adhesion number when the β_1_ subunit was blocked on the aligned collagen fibril substrate was not surprising since the β_1_ subunit is a well-characterized collagen receptor. However, it was somewhat surprising that the focal adhesion number was reduced when α_5_ (i.e., a fibronectin receptor) was blocked. It should be noted that we have previously shown that corneal fibroblasts secreted fibronectin while adhering to collagen substrates [24] and stained positively for α_5_ integrins when cultured on aligned fibrils (Figure 4). Thus, it appears that the cells also use α_5_ integrins to adhere to collagen substrates, evidenced by the blocking of this receptor subunit being accompanied by a reduction in FA number (Figure 4M).

**Figure 5:**
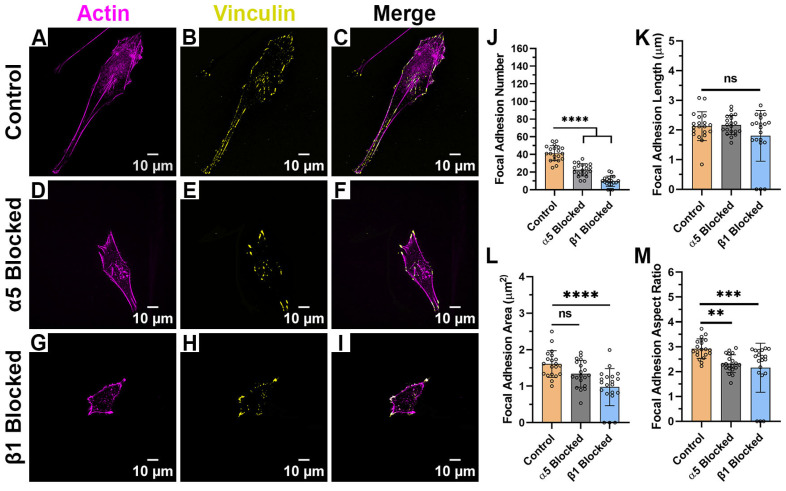
Effect of Integrin Blocking on Focal Adhesion Patterning on Random Fibril Substrates: Confocal Images for cells cultured on Random Collagen Fibril Substrates without Integrin Blocking (Control) (**A**–**C**), with α_5_ blocking antibody (**D**–**F**) and with β_1_ function blocking antibody (**G**–**I**). Quantification of Focal Adhesion morphologies with and without integrin blocking (**J**–**M**). Cells were labeled for Vinculin (Yellow) and Actin (Magenta). Scale Bar = 10 μm. Data represents Mean ± Standard Deviation for 4 experimental repeats and a total of 20 cells were analyzed. Each data point represents average values for a single cell. ns *p* > 0.05; ** *p* < 0.01; *** *p* < 0.001; **** *p* < 0.0001.

When HTKs were cultured on randomly aligned collagen fibrils (Figure 5A–I), we observed no changes in FA length (Figure 5K). However, there were significant changes in FA number, FA area and FA aspect ratio when either α_5_ or β_1_ was blocked (Table 2). Blocking the α_5_ subunit reduced the number of FAs formed by 50% (Figure 5J), the FA area by 19% (Figure 5L), and the FA aspect ratio by 21% (Figure 5M). Likewise, blocking the β_1_ subunit reduced the number of FAs formed by 77%, the FA area by 38%, and the FA aspect ratio by 28%. In addition, there were reductions in the cell area when either α_5_ or β_1_ was blocked. At this time, the exact reason why there were changes in the FA area and aspect ratio on the randomly aligned collagen fibrils and not the aligned collagen fibrils is unknown; however, we suspect this may have to do with the anisotropic nature of the aligned fibrils. In support of this are the observations that HTK cells formed fewer focal adhesions on the random fibrils in the presence and absence of blocking antibodies, as compared to the aligned collagen fibrils.

**Figure 6:**
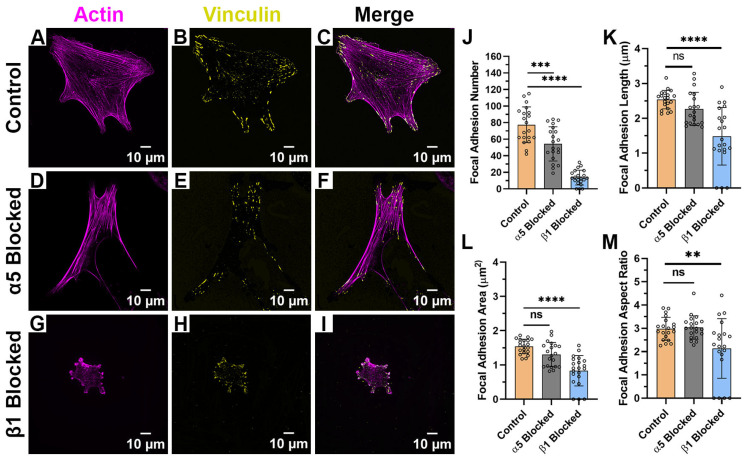
Effect of Integrin Blocking on Focal Adhesion Patterning on Monomeric Collagen-Coated Substrates: Confocal Images for cells cultured on Collagen Monomer Substrates without Integrin Blocking (Control) (**A**–**C**), with α_5_ blocking antibody (**D**–**F**) and with β_1_ function blocking antibody (**G**–**I**). Quantification of Focal Adhesion morphologies with and without integrin blocking (**J**–**M**). Cells were labeled for Vinculin (Yellow) and Actin (Magenta). Scale Bar = 10 μm. Data represents Mean ± Standard Deviation for 4 experimental repeats and a total of 20 cells were analyzed. Each data point represents average values for a single cell. ns *p* > 0.05; ** *p* < 0.01; *** *p* < 0.001; **** *p* < 0.0001.

When HTKs were cultured on monomeric collagen (Figure 6A–I), we observed significant changes in cell spreading and morphology when compared to the aligned or randomly collagen fibril substrates. In the absence (Figure 6C) and presence (Figure 6F) of the α_5_ blocking antibody, the HTKs cultured on monomeric collagen typically displayed a polygonal morphology instead of a bipolar morphology. In contrast, HTKs treated with the β_1_ blocking antibody (Figure 6I) had a significantly reduced spreading area. It is also interesting to note that the average number of focal adhesions on the monomeric collagen (~77) (Table 3) was significantly higher than on either the aligned (~54) or randomly aligned (~41) collagen fibril substrates. When the α_5_ subunit was blocked, the number of focal adhesions was significantly reduced by 30% (Figure 6J), while there was no change in the FA aspect ratio (Figure 6M). Although blocking the α_5_ subunit also reduced the FA length by 8% (Figure 6K) and the FA area by 14% (Figure 6L), these reductions were not statistically significant. Blocking the β_1_ subunit reduced the number of FAs formed by 81%, the FA length by 44%, the FA area by 44%, and the FA aspect ratio by 30%. Taken together, these results suggest that topography has a significant impact on HTK adhesion to collagen and that HTK cells primarily bind to monomeric collagen via the β_1_ receptor.

**Figure 7:**
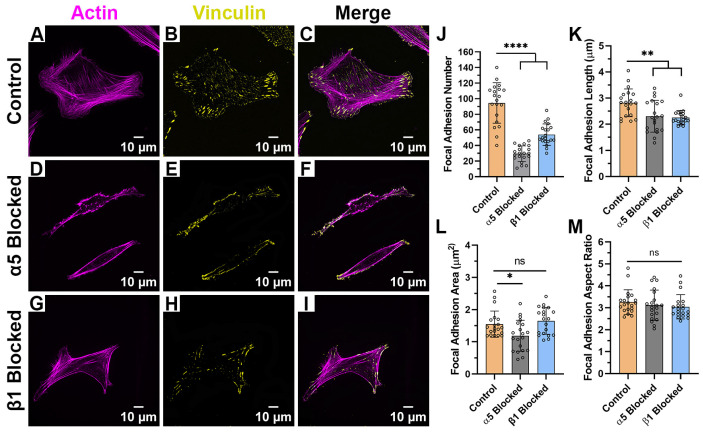
Effect of Integrin Blocking on Focal Adhesion Patterning on Fibronectin-Coated Substrates: Confocal Images for cells cultured on Fibronectin substrates without Integrin Blocking (Control) (**A**–**C**), with α_5_ blocking antibody (**D**–**F**) and with β_1_ function blocking antibody (**G**–**I**). Quantification of Focal Adhesion morphologies with and without integrin blocking (**J**–**M**). Cells were labeled for Vinculin (Yellow) and Actin (Magenta). Scale Bar = 10 μm. Data represents Mean ± Standard Deviation for 4 experimental repeats and a total of 20 cells were analyzed. Each data point represents average values for a single cell. ns *p* > 0.05; * *p* < 0.05; ** *p* < 0.01; **** *p* < 0.0001.

When HTKs were cultured on fibronectin (Figure 7A–I) in the absence of blocking antibodies, we observed cells with a polygonal morphology (Figure 7C) and an increased number of focal adhesions (Table 4) when compared to the other substrates. Blocking the α_5_ subunit significantly reduced the number of FA by 69% (Figure 7J), the FA length by 18% (Figure 7K), and the FA area by 25% (Figure 7L). Treatment of HTK cells with the β_1_ blocking antibody significantly reduced the FA number by 43% (Figure 7J) and the FA length by 18% (Figure 6K) but did not significantly change the FA area or FA aspect ratio.

### 2.5. Relative Importance of Topography and ECM Composition on Focal Adhesion Formation

**Figure 8:**
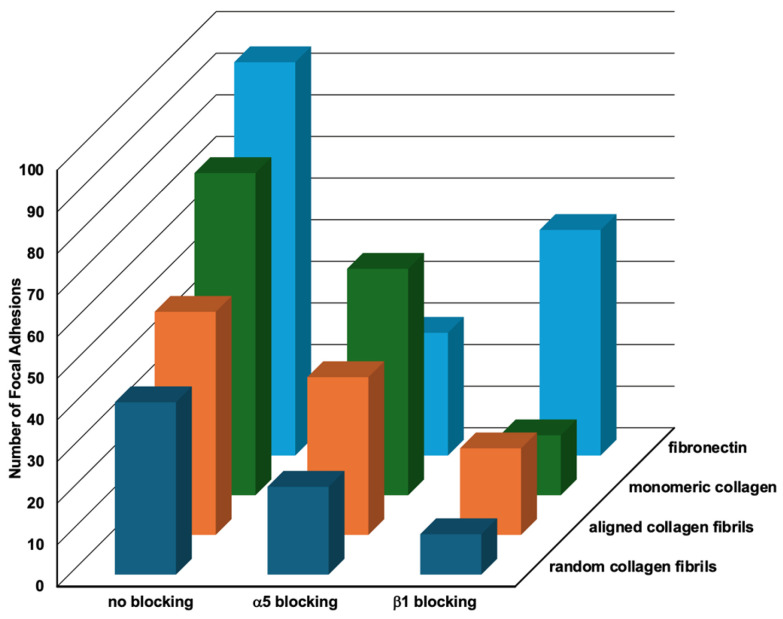
Relative Effects of Topography and Composition on Focal Adhesion Formation. Three-dimensional plot of the number of focal adhesions formed by HTK cells in the presence of different blocking antibodies when cultured on different substrate coatings (data taken from Table 1, Table 2, Table 3 and Table 4).

In order to assess the relative importance of topography vs. composition on focal adhesion formation, we plotted the data from Table 2, Table 3 and Table 4 on the number of focal adhesions formed on the different substrates in a 3D graph (Figure 8). When HTKs were cultured in the absence of a blocking antibody, they formed 22% more FAs on fibronectin as compared to monomeric collagen. Conversely, HTKs formed 31% and 47% fewer FAs on aligned collagen fibrils and random collagen fibrils than on monomeric collagen. The observation that more focal adhesions were formed on fibronectin than on monomeric collagen is consistent with a previous study that showed corneal fibroblasts adhered to fibronectin-coated substrates to a higher and stronger degree than to type I collagen and that a substrate coating of 100 μg/mL of type I collagen was required to promote attachment of approximately the same numbers of cells as attached to 10 μg/mL of FN [35]. When the α_5_ integrin subunit was blocked, HTK cells formed the most FAs on monomeric collagen, while lower numbers of FAs were formed on the substrates coated with aligned collagen fibrils, fibronectin, and random collagen fibrils. The reduction in FAs on fibronectin in the presence of the α_5_ blocking antibody was not surprising, given that α_5_ is a major fibronectin receptor. Treatment of HTK cells with the blocking antibody to the β_1_ integrin subunit resulted in a significant reduction in FA formation on all the substrates. HTKs formed the most FAs on fibronectin, followed by the aligned collagen fibrils, monomeric collagen, and random collagen fibrils. The large reduction in FAs on all three of the type I collagen substrates is consistent with the fact that the β_1_ integrin subunit is a major receptor for collagen.

To summarize the results of Figure 8, we observed that blocking of either the α_5_ or β_1_ subunits caused significant reductions in the number of FAs formed on all four substrates. It was also observed that in all three conditions, the fewest number of FAs were formed on the random collagen fibrils, and that more FAs were formed on the aligned collagen fibrils than on random collagen fibrils. It is also noteworthy that the number of FAs formed on the aligned collagen fibrils was lower than on monomeric collagen when α_5_ was blocked and lower than fibronectin when β_1_ was blocked. A possible reason for differences in FAs on the random and aligned collagen substrates might be differences in fibril diameters and the thickness of the coatings. In support of this hypothesis, there are studies that show that the orientation of the topography and fiber size can affect FA patterning and other behaviors [38,39,40]. Taken together, our results suggest that topographical cues reduce focal adhesion formation in HTK cells. While this observation is in agreement with some studies [41], other studies have reported that topographical cues increase focal adhesion formation [42]. As suggested by others, focal adhesion formation is a complex process that is dependent upon the cell type, ECM composition, and the topographical geometry [19,43,44].

## 3. Materials and Methods

### 3.1. Preparation of Microfluidic Devices and PDMS-Coated Glass Coverslips

PDMS microfluidic channels and spin-coated glass coverslips were prepared as outlined previously [23]. Briefly, Sylgard 184 Silicon Elastomer (Dow Corning; Midland, MI, USA) base and curing agent were mixed at a mass ratio of 10:1 and degassed in an AR-100 mixer (Thinky USA; Laguna Hills, CA, USA) and poured over photoresist templates. The straight line photoresist templates (width = 750 μm, height = 45 μm, length = 22 mm long) were fabricated by negative photolithography using KMPR 1050 photoresist (MicroChem, Westborough, MA, USA). After curing the PDMS at 80 °C for 1 h in an oven, the microfluidic molds were removed from the photoresist templates by cutting with an Xacto Knife. PDMS-coated glass coverslips were prepared by spin coating (1000 rpm for 30 s), degassing PDMS onto rectangular glass coverslips (45 × 50 mm, #1 thickness, Brain Research Laboratories; Waban, MA, USA), and curing overnight in a PDMS oven at 80 °C. Outlet reservoir ports were formed by punching out 2 mm diameter holes using a 2 mm Biopsy Punch (Medline Industries, Inc., Northfield, IL, USA), while inlet ports were fabricated by positioning a plastic elbow (Nordson Medical; Loveland, CO, USA) at the inlet channel of the device prior to PDMS curing.

### 3.2. Patterning of Aligned and Random Collagen Fibrils on PDMS Glass Coverslips

Aligned collagen fibrils were deposited on PDMS-coated glass coverslips by infusing chilled solutions of Type-I collagen (1.6 mg/mL) using the 750 µm wide microchannel at a 150 s^−1^ shear as previously described [23]. The 750 µm wide PDMS microchannels were reversibly bonded on the PDMS-coated glass coverslip. Chilled solutions of collagen were prepared by mixing 3 mg/mL Bovine Collagen Type-I (Advanced BioMatrix; Carlsbad, CA, USA), 0.1M NaOH, and 10X Minimal Essential Media (MEM, Life Tech; Carlsbad, CA, USA) in an 8:1:1 ratio (final collagen concentration = 1.6 mg/mL). The pH of the collagen solution was adjusted by the addition of 0.1M NaOH to a value of 7.56. To limit collagen polymerization prior to perfusion, collagen perfusion was performed in a cold room (T = 4 °C). To promote collagen fibril polymerization and deposition on the bottom PDMS surface of the microfluidic channel, the microfluidic device was placed on a hot plate set at 40 °C. Following deposition of aligned collagen fibrils, the microfluidic devices were carefully removed from the PDMS-coated glass coverslips, washed with water, and dried on the hot plate at 40 °C for 30 min. Substrates coated with random fibrils were prepared as outlined previously [23].

### 3.3. DTAF Staining and Imaging of Aligned and Random Collagen Fibrils

Aligned Collagen Fibrils were stained with 0.5% solution of (5-([4,6-Dichlorotriazin-2-yl]amino) fluorescein hydrochloride) (DTAF, Sigma-Adrich; St. Louis, MO, USA) as described previously [45]. Differential Interference Contrast (DIC) and Fluorescent Imaging of the aligned collagen fibrils was performed with a Spinning Disk Confocal Microscope (Olympus IX8, Olympus, Tokyo, Japan) with the 100X Silicone oil Apochromat objective (NA = 1.35). Quantification of collagen fibril alignment was performed using the Directionality plugin in the Image J software version 1.54g as previously described [23,45].

### 3.4. Preparation of Other ECM Coatings

To create uniform coatings of Monomeric Collagen and Fibronectin, 1 mL solutions of unpolymerized collagen (50 µg/mL) or Fibronectin (15 µg/mL) were added to the PDMS-coated coverslips. FITC labelled collagen (1 mg/mL Sigma, C4361) and Green Fluorescent Fibronectin (Fisher Scientific, NC0732510, Waltham, MA, USA) were used to coat the PDMS coverslips. The solutions were allowed to coat the substrates for 2 h at room temperature and gently washed with 1X PBS. Finally, the substrates were dried before adding the cells. For coating with poly-L-lysine (PLL), substrates were coated with 1 mL of PLL solution (Sigma P8920, St. Louis, MO, USA) for 30 min as outlined in the manufacturer’s protocol. After 30 min, the solution was removed, and substrates were allowed to dry completely for 30 min before culturing cells.

### 3.5. Cell Culture

We used an established human corneal fibroblast cell line (HTK) in which the catalytic subunit of human telomerase (h-TERT) was used to extend the lifespan of human corneal fibroblasts [46]. HTK cells were provided by the Petroll lab, and initially cultured in tissue culture flasks containing Dulbecco’s modified Eagle’s medium (DMEM) supplemented with 1% PenStrep (Invitrogen; Carlsbad, CA, USA), 0.4% sterile filtered HEPES Solution, and 10% Fetal Bovine Serum (FBS) at 37 °C in a 5% CO_2_ humidified incubator. Prior to any adhesion experiments, the HTKs were cultured for 2 days in basal (serum-free) media. HTKs were then seeded and cultured on the protein micropatterns using an established procedure [23]. Briefly, 2 mL of an HTK cell suspension (15,000 cells/mL) in basal media was added to a PDMS microwell assembly, which contained the protein micropatterned substrate and placed in a 5% CO_2_ humidified incubator at 37 °C for 24 h.

### 3.6. Antibody Blocking Experiments

To determine which integrins modulated HTK focal adhesion formation, HTKs were cultured in basal media on the protein-coated substrates for 24 h in the presence of specific integrin-blocking antibodies: mouse anti-human α_5_ integrin blocking antibody (P1D6, 25 µg/mL, Abcam ab78614) or β_1_-integrin (P5D2, 25 µg/mL, Abcam ab24693).

### 3.7. Immunofluorescence Imaging

HTK cells cultured for 24 h on the various substrates were fixed for 15 min at room temperature in a 4% paraformaldehyde (PFA) solution. The fixed cells were then washed in Phosphate Buffered Saline (PBS) three times, before being permeabilized for 15 min with a 0.5% Triton X-100 in PBS. Permeabilized cells were then blocked overnight at 4 °C in a solution of 2% Bovine Serum Albumin Fraction V (BSA Microbiological Grade Powder, Fisher Scientific, Waltham, MA, USA) in PBS. Expression of Integrin α_5_ was identified by incubating the cells with a primary antibody specific to α_5_ (Abcam ab78614, 1:100), while Integrin β_1_ was identified by incubating with a primary antibody specific to β_1_ (Abcam ab24693, 1:100) overnight at 4 °C. Cells were then washed thrice with 1X PBS and incubated for 2 h at room temperature with an Alexa Fluor 488 conjugated secondary antibody (1:200 dilution) (Invitrogen, Carlsbad, CA, USA). For visualizing focal adhesions, cells were incubated with anti-vinculin primary antibody (Sigma V284, 1:500) overnight at 4 °C. Cells were then washed thrice with 1X PBS and incubated with an Alexa Fluor TRITC conjugated secondary antibody (1:200 dilution) (Invitrogen, Carlsbad, CA, USA) for 2 h at room temperature. Finally, cells were incubated in Alex Fluor 647 Phalloidin (1:200 dilution, Molecular Probes, Eugene, OR, USA) and 4′,6-diamidino-2-phenylindole (DAPI) solution (1:1000 dilution) for 1 h overnight at 37 °C and washed 3 times in PBS. Substrates were then imaged with a 100X Silicone oil Apochromat objective (NA = 1.35) on a Spinning Disk Confocal Microscope (Olympus IX83).

### 3.8. Quantitative Analysis of Focal Adhesions

Vinculin focal adhesion quantification was performed as outlined previously [47]. ImageJ version 1.54g was used to quantify the fibrillar adhesions. For Vinculin quantification, background subtraction was used to eliminate the background, followed by thresholding. After this, the analyze particle function was used on the thresholded images to quantify the number and other morphometry parameters like area, length and aspect ratio through the ‘Set Measurements’ under the Analyze tab in Image J.

### 3.9. Statistical Analysis

Data represent the mean ± standard deviation for at least 3 independent experiments. Data were analyzed in GraphPad Prism Software 8.4.3 (GraphPad Software, Inc., La Jolla, CA, USA) using a one-way or two-way ANOVA followed by a Tukey post hoc test. A significance level of *p* < 0.05 was used for all the tests.

## 4. Conclusions

In this work, we utilized micropatterns of aligned collagen fibrils to investigate how the alignment and topography of Type I collagen fibrils and ECM composition modulate integrin expression and focal adhesions in corneal fibroblasts. Using confocal imaging and ECM proteins that are present in normal (i.e., aligned collagen fibrils) and wounded corneal stroma (i.e., random collagen fibrils, fibronectin), we observed changes in FA formation due to both the ECM composition and topography of the underlying substrate. The presence of fibrillar topography (random or aligned) resulted in diminished FA number, area, and length, while smooth coatings of monomeric collagen or fibronectin promoted more mature focal adhesions. We also observed that corneal fibroblasts formed ~50% fewer supermature focal adhesions on aligned collagen fibrils than on random collagen fibrils. Our results using blocking antibodies against specific integrin subunits confirm that human corneal fibroblasts primarily employ β_1_ integrins when adhering to collagen, while α_5_ integrin is important for adhesion to fibronectin. A surprising result was that blocking α_5_ integrin did not completely inhibit cell spreading and FA patterning when cells adhered to fibronectin. These results suggest that human corneal fibroblasts employ redundant fibronectin receptors in the absence of α_5_ integrin. It is also possible that the cells adhering to fibronectin secrete collagen and, in turn, use other collagen receptors to form fibrillar adhesions. These results highlight the need for considering topography and biochemical ligand type in designing future mechanistic studies of mechanotransduction in human corneal fibroblasts and should aid in a more rational design of biomaterials and therapies for corneal wound healing.

## Figures and Tables

**Table 1:** Focal Adhesion Morphologies on Aligned Collagen Fibril Micropatterned Substrates after Integrin Blocking.

Focal Adhesion Morphology	Control (Average ± SD)	α_5_ Blocked (Average ± SD)	β_1_ Blocked (Average ± SD)
FA Number	53.6±18.3	37.9±12.1	20.8±13.9
FA Area	1.1±0.3	1.1±0.3	0.9±0.5
FA Length	2.2±0.4	2.0±0.4	1.6±0.8
FA Aspect Ratio	3.2±0.4	2.9±0.5	2.2±1.1

**Table 2:** Focal Adhesion Morphologies on Random Fibril Substrates after Integrin Blocking.

Focal Adhesion Morphology	Control (Average ± SD)	α_5_ Blocked (Average ± SD)	β_1_ Blocked (Average ± SD)
FA Number	41.4±8.5	21.1±8.0	9.7±5.8
FA Area	1.6±0.4	1.3±0.4	1.0±0.5
FA Length	2.1±0.4	2.2±0.3	1.8±0.8
FA Aspect Ratio	2.9±0.4	2.3±0.4	2.1±0.9

**Table 3:** Focal Adhesion Morphologies on Monomer Collagen-Coated Substrates after Integrin Blocking.

Focal Adhesion Morphology	Control (Average ± SD)	α_5_ Blocked (Average ± SD)	β_1_ Blocked (Average ± SD)
FA Number	77.4±21.6	54.4±20.7	14.4±8.6
FA Area	1.5±0.2	1.3±0.4	0.8±0.4
FA Length	2.5±0.3	2.3±0.5	1.4±0.8
FA Aspect Ratio	3.0±0.5	3.0±0.5	2.1±1.3

**Table 4:** Focal Adhesion Morphologies on Fibronectin-Coated Substrates after Integrin Blocking.

Focal Adhesion Morphology	Control (Average ± SD)	α_5_ Blocked (Average ± SD)	β_1_ Blocked (Average ± SD)
FA Number	94.51±26.0	29.5±10.1	54.2±14.0
FA Area	1.6±0.4	1.2±0.5	1.7±0.4
FA Length	2.8±0.5	2.3±0.6	2.3±0.3
FA Aspect Ratio	3.3±0.6	3.1±0.7	3.0±0.6

## Data Availability

Data is contained within the article and Supplementary Materials.

## Supplementary Materials

The following supporting information can be downloaded at: https://www.mdpi.com/article/10.3390/ijms262411935/s1.
