# Supplementary material for: Effects of Topography and Extracellular Matrix Composition on Focal Adhesion Patterning in Human Corneal Fibroblasts
Source: Int J Mol Sci. 2025 Dec 11;26(24):11935. doi: 10.3390/ijms262411935 (PMC12733271; doi:10.3390/ijms262411935)
Supplement: Supplementary file 1 [file ijms-26-11935-s001.zip › ijms-3977183-supplementary.pdf]

Supplementary Data

for

Effects of Topography and Extracellular Matrix Composition on Focal Adhesion Patterning in Human Corneal Fibroblasts

Divya Subramanian [1], Nathaniel S. Tjahjono [1], Tarik Z. Shihabeddin[1], Satweka Nammi [1], Miguel Miron-Mendoza[2], Victor D. Varner [1,3], W. Matthew Petroll [2,3] and David W. Schmidtke [1,3,*]

1 Department of Bioengineering, University of Texas at Dallas, Richardson, TX 75080, USA; divya.subramanian@utdallas.edu (D.S.); nathaniel.tjahjono@utdallas.edu (N.S.T.); satweka.nammi@utdallas.edu (S.N.); vdv@utdallas.edu (V.D.V.)
2 Department of Ophthalmology, University of Texas Southwestern Medical Center, Dallas, TX 75390, USA; miguel.miron@utsouthwestern.edu (M.M.-M.); matthew.petroll@utsouthwestern.edu (W.M.P.)
3 Department of Biomedical Engineering, University of Texas Southwestern Medical Center, Dallas, TX 75390, USA
* Correspondence: david.schmidtke@utdallas.edu

Supplementary Figures

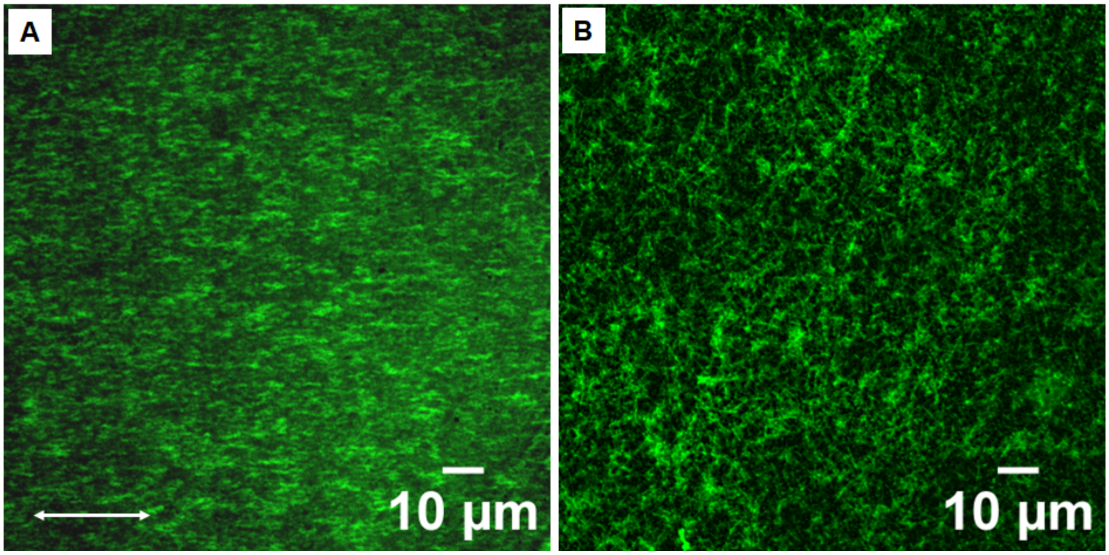

Figure S1: Fluorescent Images of aligned and random fibrils deposited on PDMS:
A) DTAF labeled Aligned Collagen Fibrils B) DTAF labeled Random Collagen Fibrils. White double arrows indicate fibril direction, and fibrils are oriented horizontally in (A). Scale Bar = 10 μm

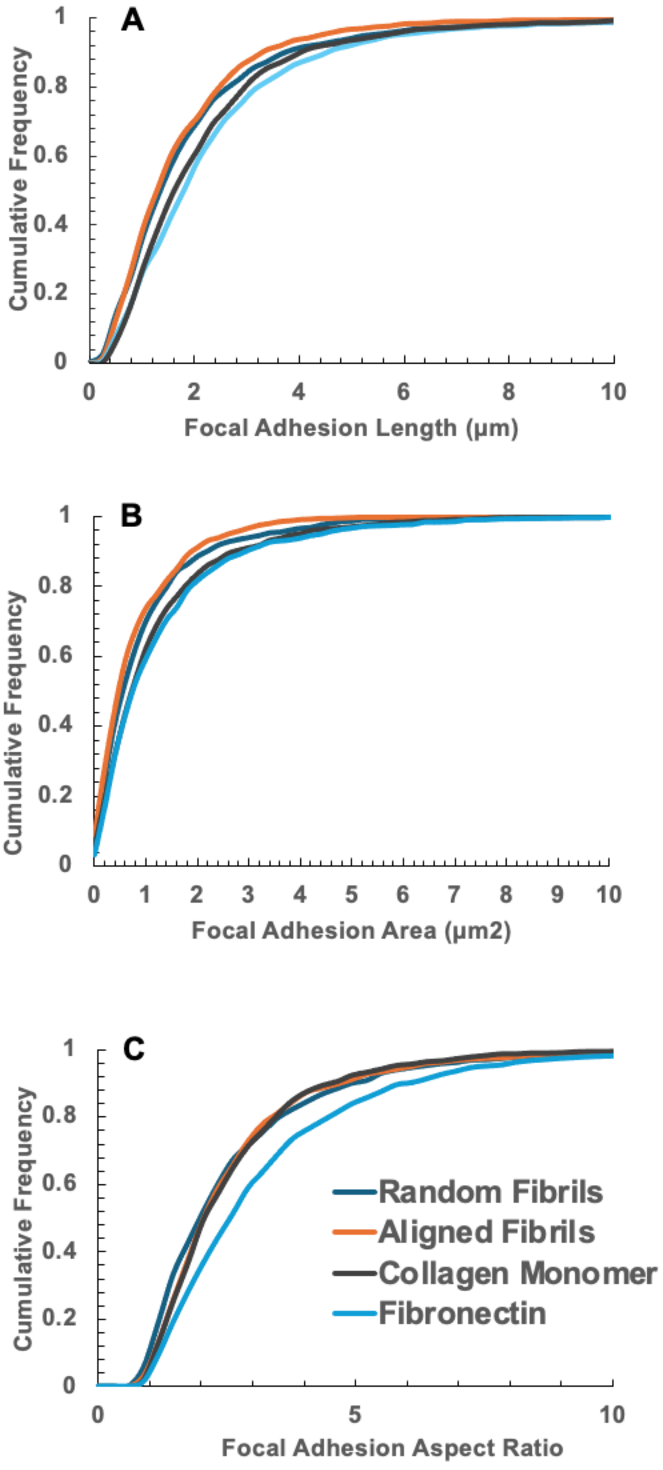

Figure S2: Cumulative Frequency Plots of Focal Adhesion Parameters for HTK cells cultured on different ECM coatings: A) Focal Adhesion Length B) Focal Adhesion Area C) Focal Adhesion Aspect Ratio. Data are plotted over 4 different repeats.
